# Gender‐Based Differences in Gut Microbiota Composition in Response to Anxiety and Stress in Shooting and Archery Athletes

**DOI:** 10.1002/brb3.70933

**Published:** 2025-09-27

**Authors:** Yuhuan Wei, Shaoye Huo, Ruyue Liang, Yuan Cui, Lihong Wang, Yunhua Zhao, Chao Zhao, Chunhai Shao

**Affiliations:** ^1^ Department of Nutrition, Shanghai Fifth People's Hospital Fudan University Shanghai China; ^2^ Shanghai University of Traditional Chinese Medicine Shanghai China; ^3^ Key Laboratory of Medical Molecular Virology, School of Basic Medical Sciences, National Clinical Research Center for Aging and Medicine, Huashan Hospital, Shanghai Medical College Fudan University Shanghai China; ^4^ Department of Nutrition, Huashan Hospital Fudan University Shanghai China

**Keywords:** anxiety, gender, gut microbiota, shooting and archery athletes

## Abstract

**Purpose:**

The purpose of this study was to investigate the prevalence of anxiety among male and female shooting and archery athletes in Shanghai, and to explore the relationships between dietary patterns, anxiety, and gut microbiota.

**Method:**

We conducted a cross‐sectional study involving 110 elite athletes. Participants were divided into four groups based on gender and anxiety status as determined by the scores of Generalized Anxiety Disorder Assessment (GAD‐7): anxious males (AM), non‐anxious males (NM), anxious females (AF), and non‐anxious females (NF). The questionnaire encompassed demographic characteristics, anxiety scales, and a 24‐h dietary recall; fecal samples were collected for 16S rRNA sequencing.

**Finding:**

Athletes in the anxious exhibited significantly higher rates of alcohol consumption, smoking, and higher education levels compared to those in the normal athletes (*p* < 0.05). Lower alpha diversity was observed in NM compared with NF (*p* < 0.05). At the phylum level, the anxious group showed a higher relative abundance of Firmicutes and a lower abundance of Actinobacteria compared to the normal group. Predictive functional profiling suggested that AM had higher endoplasmic reticulum protein processing and lower inositol phosphate metabolism, while AF displayed impaired base excision repair functionality.

**Conclusion:**

Overall, anxiety levels in elite athletes are associated with gender and gut microbial composition, suggesting that modulating the brain‐gut‐microbe axis could offer potential strategies for improving mental health and optimizing competitive performance.

## Graphical abstract

The study found that anxious athletes exhibited higher relative abundance of Firmicutes and lower levels of Actinobacteria. Predictive analyses revealed that male athletes showed increased endoplasmic reticulum protein processing, while anxious females demonstrated reduced base excision repair functions.

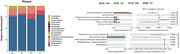

## 1 Introduction

Shooting and archery not only require athletes to possess higher precision in their competitive skills, but also often need to pay attention to the control of various kinds of stress and anxiety, since subtle disturbances in the attention process may lead to huge mistakes in performance, hence the difference in the psychological condition of professional athletes of similar level can greatly determine the outcome of the competition (Kayacan et al. 2022; Park et al. 2020). Moderate pressure is conducive to enabling athletes to perform at their true skill level, while excessive pressure and anxiety often lead to adverse reactions such as muscle tension and increased heart rate, which affects their attention, concentration and execution, and directly or indirectly affecting their performance and achievement (Sarkar and Fletcher 2014). Therefore, in‐depth understanding and research on the incidence of anxiety and stress in elite shooters and archers, the key factors affecting their anxiety, as a way to find corresponding and effective measures to reduce anxiety, are of great practical significance to improve the competitive level of this type of athletes.

Differences in anxiety between genders have been one of the topics of great interest in psychology and social sciences. Anxiety is more common in female. For example, depression and anxiety disorders are twice as prevalent in women as in men (Kundakovic and Tickerhoof 2024). Anxiety differences between genders are a complex phenomenon with multifactorial interactions, and through a deeper understanding of the mechanisms of anxiety differences between genders, we can better study mental health interventions for different gender groups.

Gut microbiota not only plays a key role in the digestive system but is also closely related to the human nervous system. In recent years, there has been increasing evidence that the gut microbiota is closely related to anxiety and depression, and differences in the diversity and abundance of the flora in anxious or depressed patients compared to the healthy population (Barandouzi et al. 2020). The microbiota‐gut‐brain axis (MGBA) is a bidirectional communication system between the gut and the brain, and gut flora disorders can affect the central nervous system (CNS) through the production of metabolites that alter the levels of neurotransmitters such as 5‐hydroxytryptophan, which ultimately contributes to the onset of depression. At the same time, many animal models of anxiety have also found that gut‐derived metabolites can cross the blood‐brain barrier and be passed between the gut and the brain, also providing evidence at the molecular level of the link between changes in gut flora and complex emotional behaviors (Cheng et al. 2024; Ma et al. 2023; Needham et al. 2022).

In the present study, we investigated the correlation with anxiety by comparing the general condition, 24‐h dietary nutrient intake, anthropometric data and fecal microbiota of male and female shooting and archery athletes.

## 2 Materials and Methods

### 2.1 Participants

This study selected participants from active shooting and archery athletes in Shanghai. Written informed consent was obtained from all participants and the possible consequences of the study were explained.

Inclusion Criteria: Shooting and archery athletes; age 12–35 years old; Han nationality; informed consent to participate in this study. Exclusion Criteria: Age < 12 or > 35 years; those who had participated in other clinical trials in the past 3 months or had taken antibiotics within 6 months; those who suffered from irritable bowel syndrome; and those who were unwilling to participate. Eventually, 110 shooting and archery athletes were included in this study, 66 males and 44 females.

### 2.2 Assessment of Anxiety Symptoms

In the survey, anxiety was first measured using the 7‐item Generalized Anxiety Disorder Scale (GAD‐7), and the total score of the GAD‐7 may range from 0 to 21 by summing up these items. The Chinese version of the GAD‐7 has shown high reliability and validity. A GAD‐7 score ≥ 7 was used as the cutoff point to screen clinical anxiety symptoms (Tong et al. 2016).

State‐Trait Anxiety Inventory‐Trait version (STAI‐T): 20 items, 10 of which are reverse scored. A 4‐point scale is used, where level 1 represents almost never; level 2 represents slightly; level 3 represents often; and level 4 represents always (Knowles and Olatunji 2020).

Competitive State Anxiety Inventory‐2 (CSAI‐2): questions 1–9 are scored as cognitive state anxiety; questions 10–18 as somatic state anxiety; and questions 19–27 as state self‐efficacy ratings. Only question 14 of is reverse scored, while the rest of the questions are normal scored (Lundqvist and Hassmen 2005).

### 2.3 Assessment of the Other Variates

Anthropometric measurements, feces collections, and food recalls were performed. General information was collected by questionnaire and anthropometric data were measured uniformly on the same day by staff. The daily energy intake of macronutrients and fiber was estimated from the 3‐day 24‐h dietary recall and calculated by dietary analysis software (Shanghai Wincome Technology Co. Ltd, China). The stool samples were stored unthawed and then transferred to the Honsunbio Technology Co. Ltd. for microbiome sequencing.

### 2.4 16S rRNA Gene Sequencing

Total DNA was extracted according to the instructions of the E.Z.N.A. Soil DNA Kit (Omega Bio‐tek, Norcross, GA, US). The concentration and purity of the DNA was checked using a NanoDrop2000 and the quality of the DNA extraction was assessed by 1% agarose gel electrophoresis. PCR of the V3‐V4 variable region was performed using primers 338F (5′‐ACTCCTACGGGGAGGCAGCAG‐3′) and 806R (5′‐GGACTACHVGGGGTWTCTAAT‐3′) to amplification.

PCR products were recovered using a 2% agarose gel, purified using the AxyPrep DNA Gel Extraction Kit (Axygen Biosciences, Union City, CA, USA) and detected on a 2% agarose gel after elution with Tris‐HCl. Quantification was performed using Qubit 4.0 (ThermoFisher, USA). The purified amplification products were constructed into Illumina libraries according to standard PCR library construction protocols and sequenced on Illumina's Miseq platform for PE300.

### 2.5 Statistical Analysis

SPSS 29.0 software was used for relevant data entry and statistical analysis. Continuous variables were presented as mean and standard deviation, while categorical variables were presented as numbers and percentages. A one‐way ANOVA was used for parametric statistical comparisons, and post hoc analysis was performed using the least significant difference (LSD) method. Effect sizes were estimated with Cohen's *d*. For categorical variables, Pearson correlation analysis was conducted to assess relationships between variables.

Prism and R Studio were used to analyze and plot the data on gut flora, to compare the differences between male and female shooting archery athletes and to explore the correlation between anxiety levels and gut flora composition. Alpha diversity was assessed using Chao1, ACE, and Shannon indices. Principal component analysis (PCA) with varimax rotation was conducted to analyze differences in microbial composition, and PERMANOVA was employed to test for differences between groups. Nonparametric rank sum tests were used to detect microbial community differences between groups. Species‐specific correlations were analyzed by Spearman correlation. *p* value of < 0.05 was considered statistically significant.

## 3 Results

### 3.1 Comparison of Basic Information and Characteristics of Athletes

**FIGURE 1:**
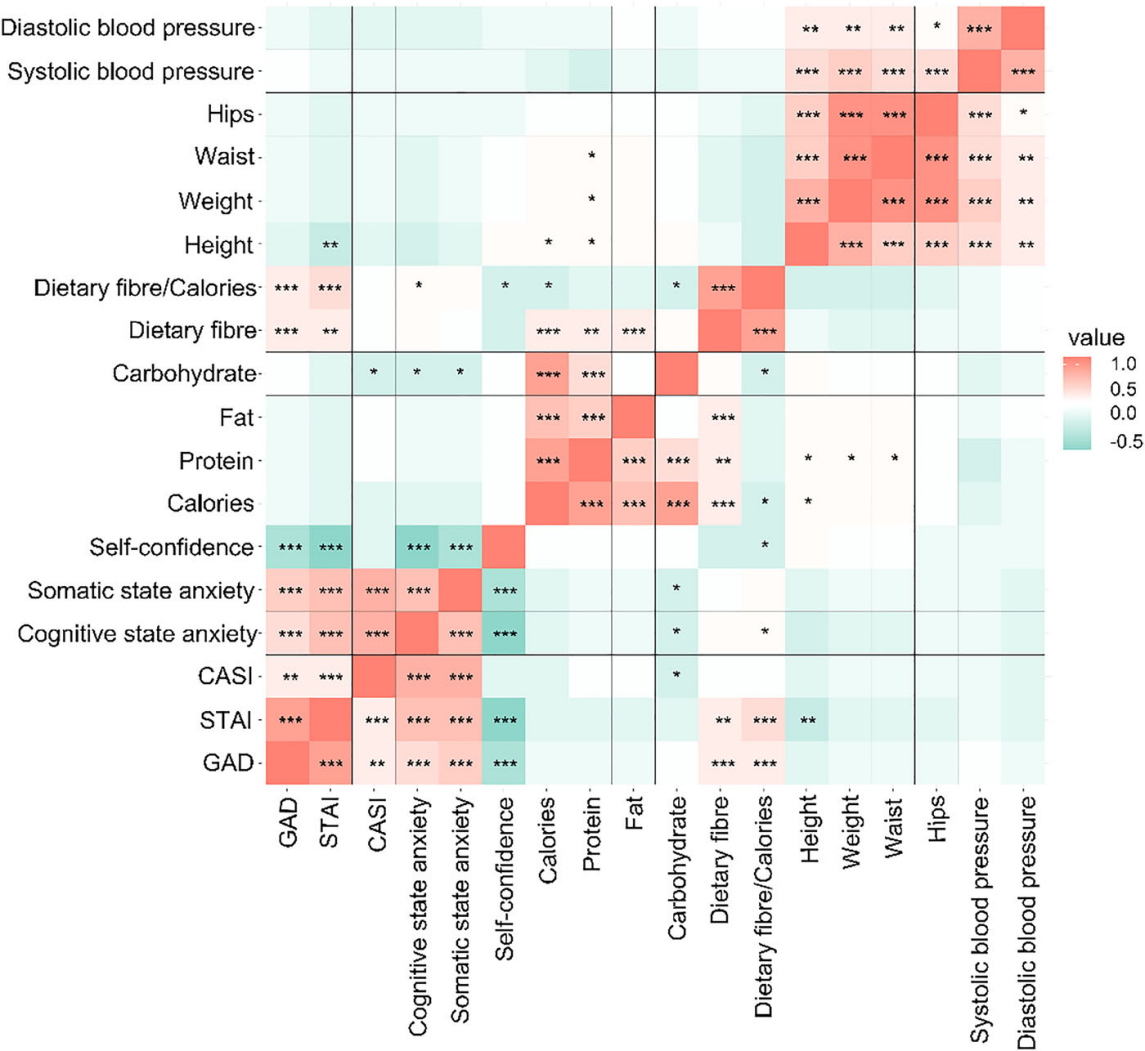
Pearson correlation analysis of anxiety scores with dietary nutrient intake and anthropometry. ^*^ *p* < 0.05, ^**^ *p* < 0.01, and ^***^ *p* < 0.001.

The overall flowchart of the study is shown in Figure 1, briefly, a total of 110 athletes, 66 males and 44 females, were included in this study. Grouping was assigned on the basis of GAD score cutoff values and gender into a normal male group of 50 and an anxious male group of 16, and a normal female group of 28 and an anxious female group of 16.

Comparison of basic information and characteristics of the four groups of athletes revealed significant differences between the groups in literacy, alcohol consumption, and smoking. The percentage of people with high school or less education in the anxious male and anxious female groups was 31.3% and 12.5%, which was significantly lower than the 44% in the normal male group and the 75% in the normal female group (*p* = 0.001). Alcohol consumption was significantly higher in 18.8% of the anxious male group than in 0% of the anxious female group (*p* = 0.020). The percentage of smokers in the anxious male group was 43.8% significantly higher than the 32% in the normal male group, and the percentage of smokers in the anxious female group was 12.5% significantly higher than the 0% in the normal female group (*p* < 0.001). The differences of the other indexes between the four groups were not statistically significant (*p* > 0.05) (Table 1).

**TABLE 1:** Sociological characteristics of the population.

Variables	NM (*n* = 50)	AM (*n* = 16)	NF (*n* = 28)	AF (*n* = 16)	*x* ^2^	*p*
Marital *n* (%)						
Married	2 (4)	0 (0)	1 (3.6)	0 (0)	1.278	0.734
Single	48 (96)	16 (100)	27 (96.4)	16 (100)		
Education *n* (%)						
Senior high school and below	22 (44)	5 (31.3)	21 (75)	2 (12.5)	22.146	0.001
Higher or post‐secondary	17 (34)	9 (56.3)	5 (17.9)	7 (43.8)		
Undergraduate and above	11 (22)	2 (12.5)	2 (7.1)	7 (43.8)		
Average monthly family income (%)						
≤ 5000	1 (2)	1 (6.3)	0 (0)	0 (0)	7.708	0.808
5001–10,000	9 (18)	5 (31.3)	4 (14.3)	3 (18.8)		
10,001–20,000	25 (50)	7 (43.8)	12 (42.9)	8 (50)		
20,001–30,000	8 (16)	1 (6.3)	4 (14.3)	2 (12.5)		
≥ 30,000	7 (14)	2 (12.5)	8 (28.6)	3 (18.8)		
Alcohol consumption (%)						
Yes	10 (20)	3 (18.8)	0 (0)	0 (0)	9.846	0.020
No	40 (80)	13 (81.3)	28 (100)	16 (100)		
Smoking (%)						
Yes	16 (32)	7 (43.8)	0 (0)	2 (12.5)	8.164	< 0.001
No	34 (68)	9 (56.3)	27 (96.4)	10 (62.5)		
Tobacco cessation	0 (0)	0 (0)	1 (3.6)	4 (25)		
Second‐hand smoking (%)						
Never	14 (28)	3 (18.8)	13 (46.4)	5 (31.3)	8.164	0.226
Sometimes	22 (44)	9 (56.3)	13 (46.3)	9 (56.3)		
Always	14 (28)	4 (25)	2 (7.1)	2 (12.5)		
Frequency of defecation (%)						
≥ Once every 3 days	2 (4)	1 (6.3)	2 (7.1)	0 (0)	11.514	0.074
Once in 1–2 days	23 (46)	7 (43.8)	22 (78.5)	9 (56.3)		
2–3 times a day	25 (50)	8 (50)	4 (14.1)	7 (43.8)		
Diarrhea (%)						
Yes	16 (32)	6 (37.5)	5 (17.9)	6 (37.5)	2.918	0.404
No	34 (68)	10 (62.5)	23 (82.1)	10 (62.5)		

Abbreviations: AF, anxiety female group; AM, anxiety male group; NF, normal female group; NM, normal male group.

### 3.2 Measurement Data and Analysis of Daily Dietary Nutrient Intake in Athletes

The results of anthropometric analysis showed that the height, weight, waist circumference, hip circumference, systolic blood pressure, and diastolic blood pressure of the normal male group were significantly higher than those of the normal female group (*p* < 0.05), and the Cohen's *d* effect sizes for those variables were higher than 0.6, and it is indicated that all the aforementioned variables exhibit medium‐to‐large effect sizes (*p* < 0.05). In addition, the height, weight, waist circumference, and hip circumference of the anxious male group were significantly higher than those of the anxious female group (*p* < 0.05). Furthermore, the Cohen's *d* effect sizes for these variables exceeded 0.8, indicating large effect magnitudes (*p* < 0.05) (Tables 2 and 3).

**TABLE 2:** Anthropometric and dietary analysis: one‐way ANOVA and LSD post hoc results.

Variables	NM (*n* = 50)	AM (*n* = 16)	NF (*n* = 28)	AF (*n* = 16)	*F*	*p*
Protein (g)	108.3 ± 41.8	113.2 ± 59.0	83.5 ± 31.0^a^	93.4 ± 36.9	2.770	0.045
Carbohydrate (g)	269.5 ± 97.4	289.6 ± 114.2	233.0 ± 94.7	261.7 ± 90.	1.453	0.232
Fat (g)	64.2 ± 27.6	70.6 ± 24.7	58.8 ± 30.2	61.6 ± 27.8	0.637	0.593
Dietary fiber (g)	7.7 ± 4.4	9.7 ± 5.0	7.8 ± 5.0	12.2 ± 5.1^b^	4.258	0.007
Dietary fiber/calories (g/kcal)	0.4 ± 0.2	0.4 ± 0.2	0.5 ± 0.3	0.7 ± 0.3^bc^	5.204	0.002
Height (cm)	178.2 ± 5.5	177.4 ± 6.1	164.7 ± 4.8^a^	167.0 ± 3.5^c^	51.660	< 0.001
Weight (kg)	75.9 ± 14.2	79.8 ± 15.1	60.0 ± 8.5^a^	62.7 ± 7.1^c^	15.222	< 0.001
Waist (cm)	84.6 ± 9.7	87.3 ± 10.1	76.1 ± 6.8^a^	76.7 ± 5.7^c^	9.721	< 0.001
Hip (cm)	99.5 ± 7.6	101.6 ± 8.3	94.5 ± 6.0^a^	95.7 ± 5.5^c^	5.022	0.003
Systolic blood pressure (mmHg)	111.4 ± 16.3	113.2 ± 13.9	100.4 ± 10.3^a^	103.8 ± 9.8	5.138	0.002
Diastolic blood pressure (mmHg)	69.2 ± 11.1	67.8 ± 10.9	61.2 ± 9.2^a^	65.6 ± 7.7	3.465	0.019

Abbreviations: AF, anxiety female group; AM, anxiety male group; NF, normal female group; NM, normal male group.

^a^ NF compared to NM, *p* < 0.05; ^b^AF compared to NF, *p* < 0.05; and ^c^AF compared to AM, *p* < 0.05.

**TABLE 3:** Effect sizes (Cohen's *d*) for group comparisons in anthropometric and dietary analysis.

Variables	Contrast	*T*	Cohen's *d*	95% CI	*P*
Protein (g)	NM vs. NF	2.733	0.645	(6.709, 42.757)	0.008^**^
	AF vs. AM	1.136	0.402	(−16.033, 55.533)	0.267
	AF vs. NF	0.948	0.297	(−11.134, 30.873)	0.348
	AM vs. NM	0.367	0.105	(−21.748, 31.521)	0.715
Carbohydrate (g)	NM vs. NF	1.712	0.404	(−5.959, 78.977)	0.091
	AF vs. AM	0.766	0.271	(−46.474, 102.261)	0.450
	AF vs. NF	1.128	0.354	(−22.685, 80.194)	0.266
	AM vs. NM	0.690	0.198	(−38.157, 78.436)	0.493
Fat (g)	NM vs. NF	0.798	0.188	(−8.048, 18.814)	0.427
	AF vs. AM	0.967	0.342	(−9.986, 27.916)	0.341
	AF vs. NF	0.298	0.093	(−15.825, 21.302)	0.767
	AM vs. NM	0.819	0.235	(−9.134, 21.302)	0.416
Dietary fiber (g)	NM vs. NF	−0.100	0.024	(−2.279, 2.060)	0.920
	AF vs. AM	−1.418	0.501	(−6.132, −1.107)	0.167
	AF vs. NF	2.785	0.873	(1.213, 7.598)	0.008^**^
	AM vs. NM	1.545	0.444	(−0.586, 4.590)	0.127
Dietary fiber/calories (g/kcal)	NM vs. NF	−1.432	0.388	(−0.194, 0.032)	0.156
	AF vs. AM	−2.298	0.812	(−0.437, 0.024)	0.030^*^
	AF vs. NF	2.119	0.664	(0.010, 0.398)	0.040^*^
	AM vs. NM	0.903	0.259	(−0.066, 0.175)	0.370
Height (cm)	NM vs. NF	10.823	2.555	(10.960, 15.903)	0.000^**^
	AF vs. AM	5.973	2.112	(6.817, 14.021)	0.000^**^
	AF vs. NF	1.594	0.500	(−0.583, 4.973)	0.118
	AM vs. NM	−0.507	0.146	(−4.042, 2.407)	0.614
Weight (kg)	NM vs. NF	6.173	1.272	(10.764, 21.019)	0.000^**^
	AF vs. AM	4.110	1.453	(8.478, 25.810)	0.000^**^
	AF vs. NF	1.057	0.331	(−2.418, 7.738)	0.297
	AM vs. NM	0.944	0.271	(−4.369, 12.193)	0.349
Waist (cm)	NM vs. NF	4.479	0.958	(4.683, 12.194)	0.000^**^
	AF vs. AM	3.647	1.289	(4.589, 16.574)	0.001^**^
	AF vs. NF	0.308	0.096	(−3.433, 4.683)	0.760
	AM vs. NM	0.978	0.281	(−2.879, 8.405)	0.332
Hip (cm)	NM vs. NF	3.009	0.710	(1.693, 8.324)	0.004^**^
	AF vs. AM	2.360	0.834	(0.791, 10.959)	0.025^*^
	AF vs. NF	0.693	0.217	(−2.420, 4.952)	0.492
	AM vs. NM	0.958	0.275	(−2.313, 6.578)	0.341
Systolic blood pressure (mmHg)	NM vs. NF	3.657	0.764	(5.027, 17.059)	0.000^**^
	AF vs. AM	2.238	0.791	(0.829, 18.171)	0.033^*^
	AF vs. NF	1.066	0.334	(−3.033, 9.819)	0.293
	AM vs. NM	0.409	0.118	(−7.182, 10.882)	0.684
Diastolic blood pressure (mmHg)	NM vs. NF	3.074	0.725	(2.671, 12.506)	0.003^**^
	AF vs. AM	0.676	0.239	(−4.551, 9.051)	0.504
	AF vs. NF	1.481	0.464	(−1.471, 9.578)	0.146
	AM vs. NM	−0.406	0.117	(−7.608, 5.038)	0.686

Abbreviations: AF, anxiety female group; AM, anxiety male group; NF, normal female group; NM, normal male group.

* *p* < 0.05

** *p* < 0.01.

According to the analysis results of daily dietary nutrient intake, the average daily protein intake of the normal male group was 108.3 g, which was higher than that of the normal female group, which was 83.5 g (*p* < 0.05, Cohen's *d* = 0.645); the average daily dietary fiber intake of the anxious female group was 12.2 g, which was higher than that of the normal female group, which was 7.8 g (*p* < 0.05, Cohen's *d* = 0.873); the dietary fiber intake per kilocalorie of the anxious female group was 0.7 g, which was higher than that of the normal female group, which was 0.5 g and the anxious male group, which was 0.4 g (*p* < 0.05, Cohen's *d* > 0.6). There were no significant differences in the average daily dietary calories, carbohydrates, and fat intake among the groups (*p* > 0.05). (Tables 2 and 3).

### 3.3 Correlation Analysis of Anxiety Scores With Dietary Nutrient Intake and Anthropometry

To explore the relationship between anxiety and its influencing factors, we analyzed anxiety scores by performing correlations with daily dietary nutrient intake and anthropometric data (Figure 1). The results showed no significant correlation between total GAD score and calorie, protein, fat, carbohydrate, and anthropometric data. However, at the same time, total GAD score was positively correlated with dietary fiber and dietary fiber to calorie ratio (*p* < 0.01), total STAI score was positively correlated with dietary fiber and dietary fiber to calorie ratio (*p* < 0.01) but negatively correlated with height (*p* < 0.01), and did not show a significant correlation with the rest of the nine items. The total CASI score showed a negative correlation with carbohydrates only (*p* < 0.05), both the somatic and cognitive scores of the CASI score showed a negative correlation with carbohydrates (*p* < 0.05), and the ratio of the somatic score to dietary fiber and calories showed a positive correlation (*p* < 0.05), but the ratio of the assertiveness score to dietary fiber and calories showed a negative correlation (*p* < 0.05).

### 3.4 Differential Analysis of the Characteristics of Intestinal Flora

**FIGURE 2:**
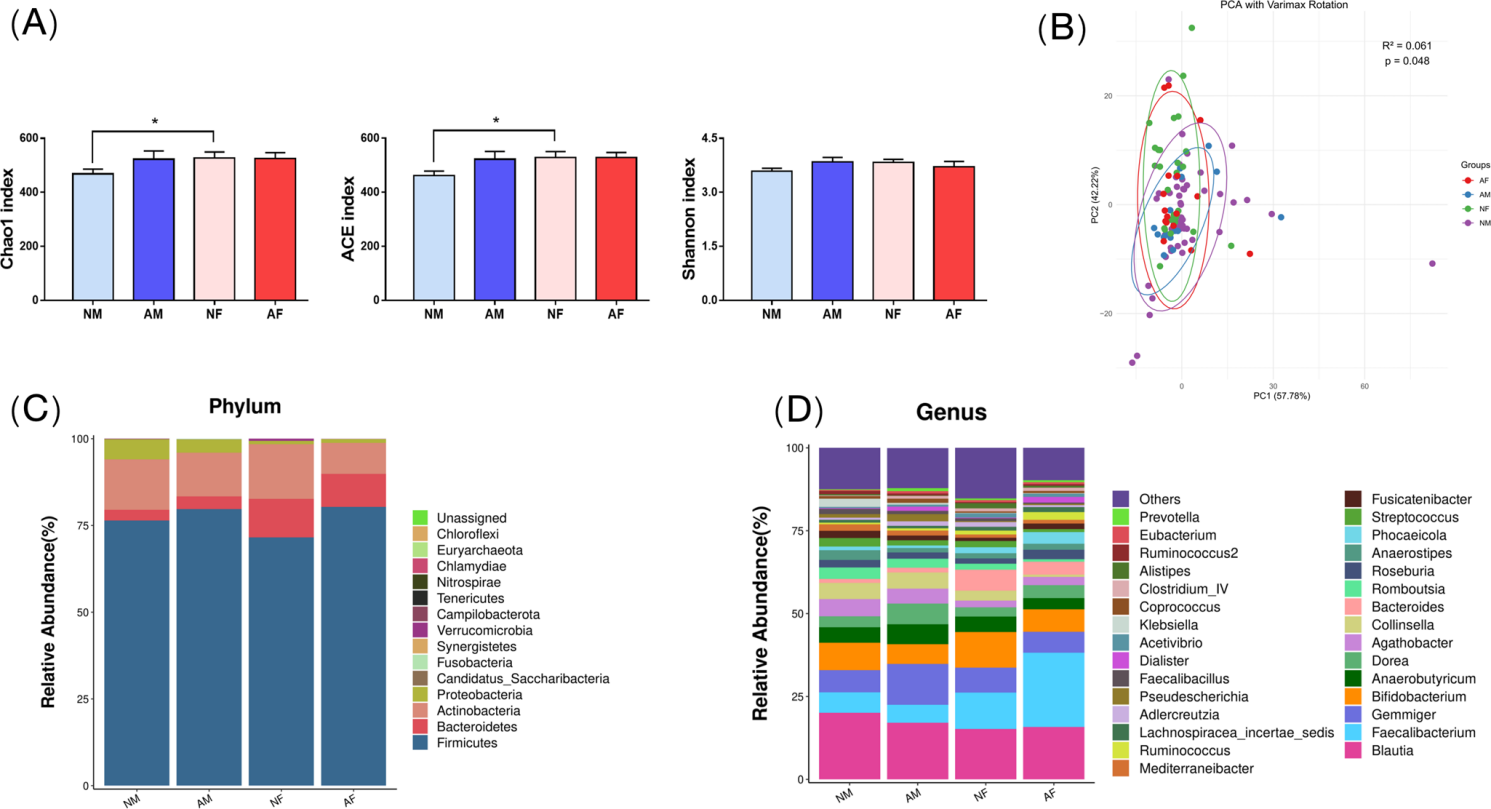
(A) Alpha diversity analysis (Chao1 index, ACE index, and Shannon index, ^*^ *p* < 0.05); (B) PCA analysis with varimax rotation; (C) relative abundance map of intestinal flora at the portal level; (D) relative abundance map of intestinal flora at the genus level. AF, anxiety female group; AM, anxiety male group; NF, normal female group; NM, normal male group.

In order to investigate the structure and composition of the gut microflora in male and female athletes with different anxiety states, we performed 16S rRNA gut flora sequencing and bioinformatic analysis. Analysis of alpha diversity based on Chao1, ACE, and Shannon found that the NF group had significantly increased alpha diversity compared to the NM group, whereas the rest of the intergroup comparisons were not significantly different (Figure 2A). PCA with varimax rotation revealed that PCA1 and PCA2 accounted for 57.78% and 42.22% of the variance in the composition of the sample communities, respectively (*p* < 0.05). These results suggest that the groups can be effectively distinguished based on these principal components, indicating significant differences in the beta diversity of bacterial populations among athletes with different genders and psychological states (Figure 2B).

To further explore the characteristics of gut flora composition in male and female athletes with different psychological states, we explored the differences in species abundance at different taxonomic levels of the gut flora in four groups of athletes. The phylum level mainly consisted of Firmicutes, Bacteroidetes, Actinobacteria, and Proteobacteria, with the largest proportion of Firmicutes and the relative abundance of Firmicutes in the anxiety group was significantly higher than that of the normal group, whereas the relative abundance of Actinobacteria showed that the anxiety group was significantly lower than the normal group (Figure 2C). At the genus level (Figure 2D), the species of intestinal flora with higher abundance were *Blautia*, *Faecalibacterium*, *Gemmiger*, and *Bifidobacterium*, respectively.

**FIGURE 3:**
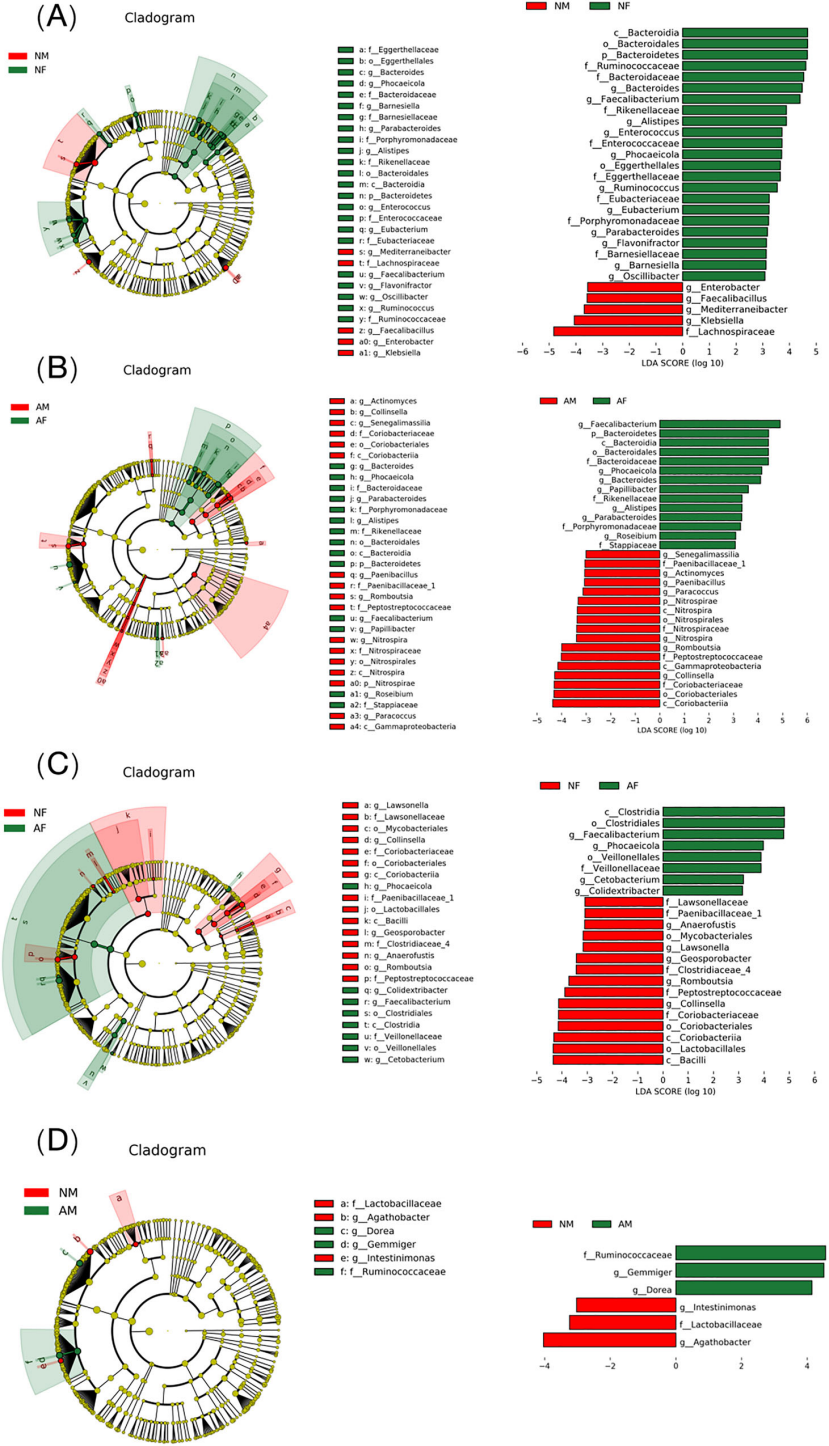
Differences in species abundance and enrichment of intestinal flora between male and female athletes with different levels of anxiety. (A) NM versus NF; (B) AM versus AF; (C) NF versus AF; (D) NM versus AM. AF, anxiety female group; AM, anxiety male group; NF, normal female group; NM, normal male group.

To explore specific differences in gut flora between male and female athletes in different psychological states, we further applied linear discriminant analysis (LDA) LefSe to identify differences in taxonomic units of flora species between groups and used LDA ≥ 3 as a threshold to identify gut flora species that were more affected by the differences. First, we analyzed the differential flora of athletes of different genders in the same psychological state. The results showed that among normal athletes, the relative abundance of 23 differential flora was higher in the female athlete group, mainly including Bacteroidia, Bacteroidales, Bacteroidetes, *Bacteroides*, Ruminococcaceae, and *Faecalibacterium*. The relative abundance of *Enterobacter*, *Faecalibacterium*, *Mediterraneibacter*, *Klebsiella*, and Lachnospiraceae was higher in the normal male group (Figure 3A). Among anxious athletes, the female anxious group showed *Faecalibacterium*, Bacteroidetes, Bacteroidia, Bacteroidales, Bacteroidaceae, Phocaeicola, and 14 other differential bacteria were more enriched, while in the male anxiety group it showed higher concentration of Coriobacteriia, Coriobacteriales, Coriobacteriaceae, *Collinsella*, Gammaproteobacteria, and 17 other differential bacteria were more enriched (Figure 3B).

We also analyzed differential genera in athletes with different levels of anxiety for the same gender. Among the female athletes, eight species of differential bacteria were predominantly found in the anxious females in the *Clostridia*, Clostridiales, *Faecalibacterium*, *Phocaeicola*, Veillonellales, and Veillonellaceae; the normal female group mainly included 15 species of differential bacteria such as Bacilli, Lactobacillales, Coriobacteriia, Coriobacteriales, and Coriobacteriaceae (Figure 3C). In male athletes: the normal male group consisted mainly of Ruminococcaceae, *Gemmiger*, and *Dorea*, and the anxious male group was dominated by *Intestinimonas*, Lactobacillaceae, *Agathobacter* (Figure 3D).

### 3.5 Correlation Analysis

**FIGURE 4:**
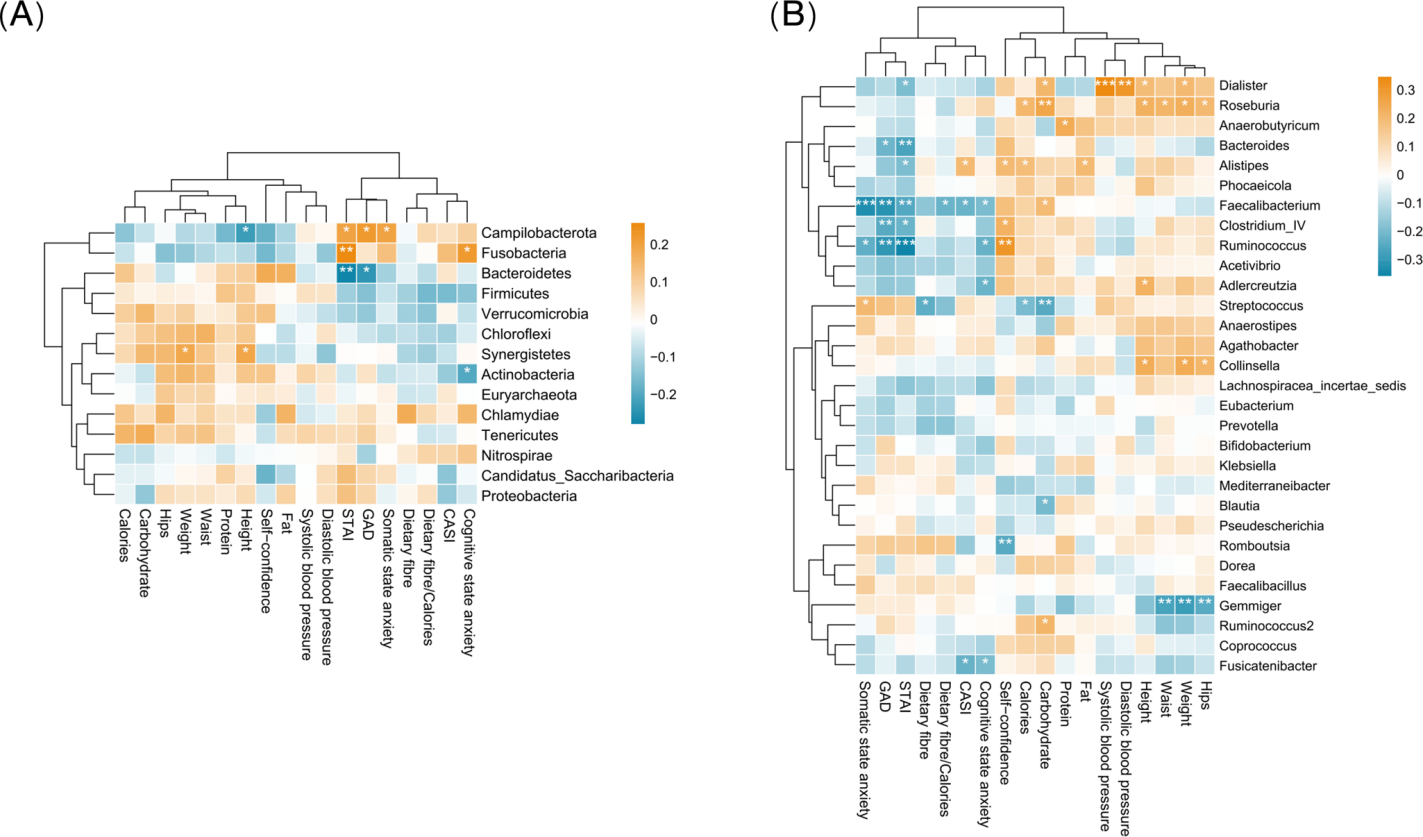
Heat map of correlation analysis between gut flora and anxiety level, daily dietary nutrient intake and anthropometric data. (A) Correlation between gut flora and anxiety level, daily dietary nutrient intake and anthropometric data at the phylum level. (B) Correlation between gut flora and anxiety level, daily dietary nutrient intake and anthropometric data at the genus level. ^*^ *p* < 0.05, ^**^ *p* < 0.01, and ^***^ *p* < 0.001.

Pearson correlation analyses demonstrated the correlations between the phylum‐level flora and the top 30 genus‐level flora in terms of abundance with anxiety levels, daily dietary nutrient intake, and anthropometric measurements (Figure 4A,B). This correlation analysis revealed that Campylobacterota was significantly positively correlated with STAI, GAD, and somatic anxiety state, while it was significantly and negatively correlated with height. Fusobacteria showed a significant positive correlation with cognitive state anxiety and STAI, whereas Bacteroidetes showed a significant negative correlation with STAI and GAD, and Actinobacteria showed a significant negative correlation with cognitive state anxiety. The Synergistetes showed significant positive correlation with height and weight (Figure 4A). None of the remaining phylum level flora showed significant correlation.

At the genus level, *Dialister* was significantly and positively correlated with carbohydrate, systolic blood pressure, diastolic blood pressure, height, and weight (Figure 4B). *Roseburia* was significantly and positively correlated with calories, carbohydrates, height, weight, waist, and hips. *Bacteroides* showed significant negative correlation with GAD and STAI. *Alistipes* showed significant positive correlation with CASI, self‐confidence, carbohydrate, and fat intake but showed significant negative correlation with STAI. And *Fusicatenibacter* showed significant negative correlation with CASI and cognitive state anxiety. Both *Clostridium_IV* and *Ruminococcus* showed significant negative correlations with GAD and STAI, but positive correlations with self‐confidence, while *Ruminococcus* also showed significant negative correlations with cognitive state anxiety and somatic state anxiety. *Streptococcus* showed significant positive correlation with somatic state anxiety but significant negative correlation with dietary fiber, calories, and carbohydrates, whereas *Faecalibacterium* showed significant negative correlation with STAI, GAD, CASI, somatic state anxiety, cognitive state anxiety, and dietary fiber to calories ratio, and significant positive correlation with carbohydrates. *Collinsella* showed significant positive correlation with height, weight, and hips, *Romboutsia* showed significant negative correlation with self‐confidence. *Adlercreutzia* was positively correlated with height and *Gemmiger* was negatively correlated with waist, weight, and hips. *Ruminococcus2* showed significant positive correlation with carbohydrates and *Blautia* showed significant negative correlation with carbohydrates.

### 3.6 Differential Function Prediction of Intestinal Flora

In order to understand the functional differences in gut flora between athletes of different genders and different levels of anxiety, we performed functional prediction analyses of gut flora between groups.

**FIGURE 5:**
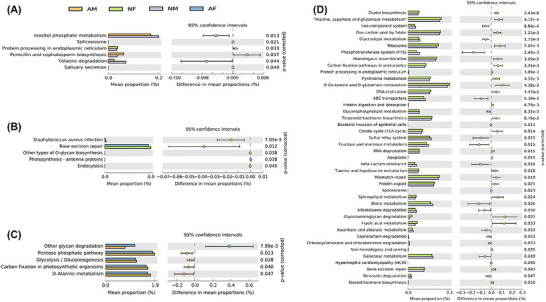
Comparative analysis of functional prediction between groups. (A) NM vs AM; (B) NF vs AF; (C) AM vs AF; (D) NM vs NF. AF, anxiety female group; AM, anxiety male group; NF, normal female group; NM, normal male group.

In male athletes, the functional abundance of protein processing in endoplasmic reticulum and penicillin and cephalosporin biosynthesis genes in the gut flora of the anxiety group was significantly higher than that of the normal group, while the relative abundance of inositol phosphate metabolism and toluene degradation function genes was significantly lower than in the normal group (Figure 5A); in female athletes, the abundance of *Staphylococcus aureus* infection and base excision repair (BER) function in the intestinal flora of the anxiety group was significantly lower than in the normal group (Figure 5B).

We also compared functional differences in gut flora between genders. Functional abundance of other glycan degradation was found to be significantly higher in the gut flora of females than males in anxious athletes, whereas functional abundance of pentose phosphate pathway, glycolysis/gluconeogenesis, carbon fixation in photosynthetic organisms, and *D*‐alanine metabolism was significantly lower than in males (Figure 5C). In normal athletes, between male and female zeatin biosynthesis, alanine, aspartate, and glutamate metabolism, two‐component system, glycerolipid metabolism, ribosome, phosphotransferase system, homologous recombination, carbon fixation pathways in prokaryotes showed significant differences (Figure 5D).

## 4 Discussion

This study investigated the basic information, anthropometric data, and 24‐h dietary nutrient intake of 110 active elite athletes from the Shanghai Shooting and Archery Sports Management Center, and collected fecal samples from the athletes for intestinal flora for 16S rRNA sequencing. By grouping athletes according to anxiety scale scores and gender, we compared basic profiles, anthropometric measurements, dietary intake, and gut flora diversity and relative abundance between them as a means of investigating the interrelationships between anxiety and gut flora with factors such as gender.

In comparative analysis of basic demographic information, we found that higher educational attainment, alcohol consumption, and smoking may be factors that influence anxiety. Among them, highly educated people may be more likely to be at increased risk of anxiety and depression due to high demands on themselves, higher work pressure, and social expectations. Of course, poor lifestyle, smoking, and drinking can also affect mental health, with chronic nicotine intake and alcohol abuse leading to an increase in anxiety‐like behaviors (Casarrubea et al. 2020; Socodato et al. 2020).

Previous research has shown a potential link between the composition of gut microbes and dietary patterns and depression and anxiety (Y. Yao et al. 2023). Food is strongly correlated with gut flora, and microbes influence host immunity and metabolism and have a wide range of effects on mental health. Unhealthy dietary habits have been linked to psychological states such as anxiety; for example, people who are addicted to regular fried foods and milk tea have a higher risk of developing anxiety and depression (Qu et al. 2023). And there is also a correlation between dietary fiber consumption and anxiety. Chen et al. conducted a randomized controlled trial with an 8‐week intervention in the high‐fiber diet group, which showed that high‐fiber dietary intake reduced systemic inflammation, increased the number of beneficial bacteria and reduced the number of harmful bacteria (Chen et al. 2023). In addition, patients who received the high‐fiber diet intervention had a better mood state and reduced symptoms of depression and anxiety. Another study demonstrated that high dietary fiber intake increased short‐chain fatty acid (SCFA) production, while improving cognitive performance and anxiety‐like behaviors in postnatal stress mice (Z. Liu et al. 2020). However, some studies have also found that vegetarianism is associated with increased anxiety and depression among Chinese students (Lavallee et al. 2022). This study revealed a significantly positive relationship between dietary fiber intake and anxiety. However, this result may be influenced by the sample size, as well as the types and varieties of dietary fiber consumed in this study. Therefore, further research and investigation are still needed. Furthermore, we found that carbohydrates showed a significant negative correlation with anxiety, which is consistent with the findings of others (S. Yao et al. 2022). This implies that there are correlations between nutrients such as carbohydrates and dietary fiber and anxiety, and that further alleviation of anxiety by improving dietary patterns and dietary intake deserves to be confirmed by more studies in the future.

The intestinal flora is a type of microorganism community and plays a key role in maintaining intestinal health and normal functional operation. As the main dominant bacteria constituting the human intestinal flora, the Firmicutes and Bacteroidetes (F/B) contribute to the maintenance of homeostasis in the individual's internal environment through nutritional, immunomodulatory, and systemic inflammatory pathways (J. Liu et al. 2022; Sun et al. 2023). In addition, the ratio of the F/B and *Prevotella* has been associated with obesity (Aragon‐Vela et al. 2021), but there are conflicting conclusions about the effects of the F/B on anxiety. Our study showed that Firmicutes were more abundant in anxious athletes, whereas Actinobacteria were more abundant in normal athletes. In addition, female athletes were more abundant in Bacteroidetes than males. Also, in the correlation analysis, we concluded that Bacteroidetes were positively correlated with anxiety, while Fusobacteria were negatively correlated with anxiety. It has been shown that increasing the abundance of beneficial bacteria such as *Lactobacillus* and Firmicutes in the gut while decreasing the abundance of detrimental bacteria such as Proteobacteria, *Bacteroides*, and Actinobacteria is effective in alleviating depression and anxiety‐like behavior in mice (Lan et al. 2024). It has also been shown in the literature that an increase in the abundance of Actinobacteria and a decrease in the abundance of Firmicutes are usually observed in depressed patients (McGuinness et al. 2022). Although all contrary to the findings of the present study, it has also been shown in the literature that the gut microbiota of patients with anxiety and depression contains fewer Firmicutes and more Actinobacteria than in the healthy population (Bastiaanssen et al. 2020; Li et al. 2022). Surface polysaccharides synthesized by *Bacteroides* are involved in host lymphocyte regulation and cytokine expression and play an important role in the prevention of tumors, colitis, and atherosclerosis (Jiang et al. 2017; Mazmanian et al. 2008; Ulsemer et al. 2013). However, unlike probiotics in the traditional sense, the effects of *Bacteroides* on human health are affected by multiple aspects, and some *Bacteroides* can also lead to the development of a number of diseases under different environments, which can seriously threaten human health (Salyers et al. 2004; Shoemaker et al. 2001). The Firmicutes have a system for degrading dietary fiber, but unlike the Bacteroidetes, the Firmicutes prefer to transport dietary fiber intracellularly for degradation, while the beneficial bacteria of the Firmicutes have a great potential in the treatment of metabolic and inflammatory diseases, controlling systemic immune system function (Sender et al. 2016).

At the genus level, the gut flora of anxious athletes contained more *Dialister*, *Acetobacter*, and *Dorea*, whereas normal athletes contained more *Bifidobacterium* and *Streptococcus*. Also, in the correlation analysis, we concluded that *Bacteroides*, *Actinobacteria*, *Clostridium, Ruminococcus, Faecalibacterium*, *Fusicatenibacter* were positively correlated with anxiety, whereas Campylobacterota, *Streptococcus*, and *Romboutsia* were negatively correlated with anxiety. It has been shown that *Coprococcus* and *Dialister* are scarcer in the intestines of depressed populations, and that reduced abundance of individual *Ruminococcus* is associated with increased depressive symptoms, which is different from the results of the present study, possibly due to the small sample size (Chin Fatt et al. 2023; Valles‐Colomer et al. 2019). In addition, some studies have found that *Bacteroides thetaiotaomicron* and *Clostridium difficile* are more prevalent in depressed female patients (Li et al. 2023). Consumption of *Bifidobacterium*‐rich probiotics has been associated with reduced stress and improved memory, as well as reduced negative emotions such as anxiety and depression (Allen et al. 2016).

Microorganisms and their metabolites in the gut can also produce a variety of neurotransmitters such as serotonin, 5‐hydroxytryptamine (5‐HT), which affects mood and behaviors (O'Mahony et al. 2015). Two probiotics, *Bifidobacterium* and *Lactobacillus* have been found to increase the availability of the serotonin precursor tryptophan, which in turn elevates serotonin. Studies have shown that imbalances in γ‐aminobutyric acid (GABA) metabolism may play a role in the pathogenesis of depression (Chevalier et al. 2020; Jenkins et al. 2016). GABA is a CNS inhibitory neurotransmitter present in a large number of mammals, and it has been closely associated with ENS function and CNS disorders (Strandwitz 2018). In addition, SCFAs (butyric, acetic, and propionic acids, among others) produced by fermentation of intestinal flora directly influence emotional symptoms such as host anxiety (Parada Venegas et al. 2019). Butyric acid is a bridge between gut microbes and host metabolism that promotes intestinal regulatory T cell differentiation and production of anti‐inflammatory cytokines (Burokas et al. 2017; Furusawa et al. 2013). It has been shown that the abundance of butyrate‐producing fecal cocci and fecal bacilli decreases with the onset of depression, while the prevalence of depression is higher among individuals with low abundance of intestinal anaplasmosis, in addition to alterations in anxiety and anxiety‐depression‐like behaviors correlating significantly with alterations in SCFA levels in mice (Valles‐Colomer et al. 2019). This suggested that intestinal flora and their metabolites have an important role in reducing anxiety and enhancing athletes’ performance. The dynamic changes in the levels of relevant hormone indicators and neurotransmitters in the serum of athletes, as well as how to achieve this effect through dietary intake or the improvement of safety‐grade functional health food, are the directions of our future exploratory research and application.

Sex differences play a crucial yet often overlooked role in the MGBA, influencing its structure and function at multiple levels. Sex steroids shape the composition of the gut microbiota, which in turn regulates levels of bioactive sex hormones, thereby playing a key role in the regulation of mental health and overall functions (Hokanson et al. 2024). Previous studies have shown that sex differences in microbial alpha diversity evolve over the lifespan, peaking from adolescence to middle age, with females typically exhibiting higher diversity (de la Cuesta‐Zuluaga et al. 2019). Consistently, our study found that female athletes have significantly higher gut microbiota diversity than males, suggesting sex may influence microbial richness through hormonal or lifestyle factors. Studies have shown that chondroitin sulfate and its oligosaccharides promote a dependence on *Bacteroides* for polysaccharide metabolism in female mice (Shang et al. 2016). Similarly, this study found that female athletes have a higher abundance of *Bacteroides*, which may be related to their higher intake of dietary fiber/calories. Previous studies have identified Lachnospiraceae as potential biomarkers for alcohol‐induced dysbiosis in the gut microbiota (Brigagao Pacheco da Silva et al. 2025). In this study, the higher abundance of these taxa in male athletes may be associated with their more frequent alcohol consumption. Research indicates that early‐life gut inflammation can induce alterations in microbiota composition, closely associated with the regulation of SCFAs and androgen levels (Sullivan et al. 2025). In addition, studies have shown that a high‐fat diet in male mice promotes an increase in certain uncultured *Collinsella aerofaciens*‐related bacteria, exhibiting sex‐specific effects and potential pro‐inflammatory roles (Hases et al. 2023). In our study, we observed a significant elevation of *Collinsella* in male patients with anxiety, suggesting its possible involvement in inflammatory responses and the development of anxiety symptoms. These findings highlight the crucial role of sex‐related gut microbiota changes and inflammation in the regulation of anxiety and neuropsychiatric disorders, with important implications for clinical interventions.

In the prediction of gut flora function, we concluded that in male anxiety athletes, the abundance of endoplasmic reticulum protein processing in the gut flora was significantly higher than in the normal group, whereas the abundance of phosphatidylinositol metabolism was significantly lower than in the normal group. In female athletes, the abundance of *Staphylococcus aureus* infection and BER function in the gut flora was significantly lower in the anxiety group than in the normal group. Endoplasmic reticulum protein processing is most notably glycosylation, and excessive sugar intake leads to the production of advanced glycation end‐products (AGEs), which affect brain function, induce, and exacerbate the development of psychiatric symptoms, and are one of the culprits of chronic inflammation (Hirai et al. 2021; Uribarri et al. 2015). Meanwhile, Fan et al. revealed that astrocytes in the endoplasmic prefrontal cortex are involved in the regulation of depressive‐like behaviors, and that lowering the level of glycosylation modifications can reduce stress‐induced disorders of glutamate metabolism, which in turn protects the neuronal synaptic structure and function, and produces an antidepressant effect (Fan et al. 2023). Inositol phosphate, as a metabolite of intestinal flora, can activate histone deacetylase (HDAC) in our body to promote intestinal epithelial repair, which is conducive to the maintenance of a healthy state of the organism, and therefore inositol has been used to treat symptoms of depression and anxiety (Mukai et al. 2014; Thomas et al. 2016; Wu et al. 2020). Evidence from previous studies describes that patients with bipolar disorder exhibit a depressive state with low levels of inositol in the CNS, and that the most commonly used mood stabilizers may act on inositol metabolism by inducing myo‐inositol depletion, thereby inhibiting overactive signaling (Campbell et al. 2022). BER is a first‐line repair pathway that maintains the genetic stability of the organism, and it is effective in repairing a wide range of DNA damage types. DNA is important for healthy aging and coping with stress (Yang et al. 2019). It has been shown that accumulation of unrepaired DNA damage in the brain alters GABA neurotransmission and leads to congenital and acquired anxiety‐related behavioral deficits in a gender‐dependent manner (Mueller et al. 2022). These above findings are consistent with those of the present study.

### 4.1 Limitations

The present study also has some limitations in that, in addition to its inherent inability to determine causality as a cross‐sectional study, it did not delve into the mechanistic relationship between anxiety and gut microbiota. Fortunately, other relevant literature may provide more details on this mechanistic relationship, thus compensating for the shortcomings in this regard. In the future, we will also increase the sample size and collect in‐depth data such as serum hormone indicators to further improve the study.

## 5 Conclusion

In this study, we found that anxiety levels in elite shooting and archery athletes were closely related to gender and specific microbial community structures in the gut. Modulating the brain‐gut‐microbe axis may be a new way to promote athletes’ mental health and enhance competitive performance in the field of sports medicine.

## Author Contributions

Chunhai Shao designed the study and obtained funding. Chao Zhao participated in the design of the study, reviewed, and edited the manuscript. Yuhuan Wei, Shaoye Huo and Ruyue Liang also designed the study, performed the statistical analysis, prepared the figures, and wrote the draft. Yuan Cui, Lihong Wang and Yunhua Zhao participated in the data collection. Yuhuan Wei polished this manuscript. Yuhuan Wei, Shaoye Huo and Ruyue Liang are considered co‐first authors. Chao Zhao and Chunhai Shao are considered co‐correspondence authors. All authors contributed to the article and approved the submitted version.

## Ethics Statement

This study involves human participants and was approved by the Ethics Committee of Shanghai Fifth People's Hospital, Fudan University, with all participants providing written informed consent (Approval No. 2020002X1).

## Conflicts of Interest

The authors declare no conflicts of interest.

## Peer Review

The peer review history for this article is available at https://publons.com/publon/10.1002/brb3.70933.

## Data Availability

The original contributions presented in this study are included in the article; further inquiries can be directed to the corresponding authors.
